# TAK1 Is Required for Survival of Mouse Fibroblasts Treated with TRAIL, and Does So by NF-κB Dependent Induction of cFLIPL

**DOI:** 10.1371/journal.pone.0008620

**Published:** 2010-01-08

**Authors:** Josep Maria Lluis, Ulrich Nachbur, Wendy Diane Cook, Ian Edward Gentle, Donia Moujalled, Maryline Moulin, Wendy Wei-Lynn Wong, Nufail Khan, Diep Chau, Bernard Andrew Callus, James Edward Vince, John Silke, David Lawrence Vaux

**Affiliations:** 1 Deparment of Biochemistry, La Trobe University, Bundoora, Australia; 2 School of Biomedical, Biomolecular and Chemical Sciences, University of Western Australia, Crawley, Australia; 3 Department of Biochemistry, University of Lausanne, Epalinges, Switzerland; University of Texas MD Anderson Cancer Center, United States of America

## Abstract

Tumor necrosis factor (TNF)-related apoptosis-inducing ligand (TRAIL) is known as a “death ligand”—a member of the TNF superfamily that binds to receptors bearing death domains. As well as causing apoptosis of certain types of tumor cells, TRAIL can activate both NF-κB and JNK signalling pathways. To determine the role of TGF-β-Activated Kinase-1 (TAK1) in TRAIL signalling, we analyzed the effects of adding TRAIL to mouse embryonic fibroblasts (MEFs) derived from TAK1 conditional knockout mice. TAK1−/− MEFs were significantly more sensitive to killing by TRAIL than wild-type MEFs, and failed to activate NF-κB or JNK. Overexpression of IKK2-EE, a constitutive activator of NF-κB, protected TAK1−/− MEFs against TRAIL killing, suggesting that TAK1 activation of NF-κB is critical for the viability of cells treated with TRAIL. Consistent with this model, TRAIL failed to induce the survival genes cIAP2 and cFlipL in the absence of TAK1, whereas activation of NF-κB by IKK2-EE restored the levels of both proteins. Moreover, ectopic expression of cFlipL, but not cIAP2, in TAK1−/− MEFs strongly inhibited TRAIL-induced cell death. These results indicate that cells that survive TRAIL treatment may do so by activation of a TAK1–NF-κB pathway that drives expression of cFlipL, and suggest that TAK1 may be a good target for overcoming TRAIL resistance.

## Introduction

TRAIL is a member of the tumor necrosis factor superfamily that selectively induces apoptosis in a wide variety of cancer cells, while sparing normal cells, highlighting its potential as an agent for cancer therapy[1]. So far, the mechanism for differential TRAIL sensitivity has not been established.

Murine TRAIL is known to bind to three different receptors: mTRAIL-R which contains a death domain (DD) in the intracellular portion, and mDcTRAIL-R1 and mDcTRAIL-R2, which are decoy receptors that regulate the binding of TRAIL to mTRAIL-R[2].

TRAIL triggers apoptosis by binding to mTRAIL-R, which leads to the recruitment of Fas associated death domain (FADD) through its DD. The adaptor protein FADD also contains a death effector domain (DED) that allows the binding of inactive procaspase 8 and cellular FLICE-inhibitory protein (cFlip). Once this death-inducing signalling complex (DISC) has been assembled, self-cleaved caspase 8 will lead to the activation of effector caspases 3 and 7 resulting in apoptotic cell death.

cFlip is the only protein present in the mTRAIL-R DISC that is capable of blocking death receptor-mediated apoptosis. In mouse cells, cFlip exists mainly in three forms: cFlipL and cFlipR that arise from mRNA splicing, and the cleaved form, Flipp43 [3], [4]. All these variants of cFlip bear two DED domains but only cFlipL possesses a caspase-like domain, which lacks catalytic activity. Therefore, all cFlip forms are potentially able to compete with procaspase 8 for binding to the DED of FADD, preventing its full activation and, thereby, cell death. Interestingly, elevated levels of cFlip protein have been reported in different types of cancer [5], [6], [7], [8], and cFlip gene silencing can sensitize tumor cells to TRAIL induced cell death in many cases[9], [10], [11], [12], [13].

While apoptosis is the major outcome for many types of cancer cells exposed to TRAIL, there is accumulating evidence that TRAIL can also activate NF-κB and c-Jun N-terminal kinase (JNK) pathways [14], [15], [16]. The effects of NF-κB and JNK on TRAIL signalling are controversial, with some reports showing that their activation protects cells from TRAIL induced apoptosis [17] and others suggesting the opposite effect [18]. Activation of NF-κB by TRAIL is of particular interest, because of its ability to induce anti-apoptotic genes such as cFlip, cIAPs, A20, and Mcl-1[19], [20].

Although complexes that transmit signals from TRAIL receptors have not been thoroughly characterised, subsequent to assembly of TRAIL DISC it has been reported that a secondary complex is formed containing FADD, TNF receptor-associated death domain (TRADD), receptor interacting protein (RIP1), TNF receptor associated factor 2 (TRAF2) as well as IKKγ, and this is crucial for NF-κB and JNK activation by TRAIL [15], [21].

On the other hand, TAK1, a member of the MAP3K family, was originally identified as a kinase involved in TGF-β signalling. TAK1 is activated by a wide range of cytokines such as TLR, IL-1 and TNF [22]. Activated TAK1 then is able to phosphorylate IKK and MKK, leading to the activation of NF-κB and JNK [23]. Recently, TAK1 has been shown to be involved in survival of cells treated with TRAIL [24], [25], [26] but there are discrepancies between the cellular mechanisms postulated to explain how TAK1 determines TRAIL sensitivity.

Here, we show that the kinase activity of TAK1 is required for transformed mouse fibroblasts to survive treatment with TRAIL. Although TRAIL induced JNK and NF-κB activation was abolished in the absence of TAK1, only NF-κB appears to play a key role in allowing survival of TRAIL treated cells. Interestingly, NF-κB dependent induction of cFlipL in TAK1 knockout MEFs was able to inhibit TRAIL killing. Thus, we propose that TRAIL induced cFlip upregulation, signalled through TAK1 and NF-κB, is essential for transformed mouse fibroblasts to survive in the presence of TRAIL.

## Materials and Methods

### Ethics Statement

All mouse work was done according to the requirements of La Trobe University Animal Ethics Committees with ethics approval number: AEC 09-01-B.

### Constructs, Antibodies, and Reagents

Cre-recombinase and SV40 Large T antigen were cloned into lentiviral vector pFU. In the tamoxifen inducible lentiviral system, the inducible transcriptional activator Gal4 1-147 ER^T2^ VP16 (GEV16) was cloned into pFU PGK Hygro, and the genes TAK1, TAK1(K63W), IKK2EE, cFlipL, cFlipp43, cFlipR were cloned into pF 5x UAS SV40 Puro vector. Murine cIAP2 was cloned in doxycycline regulated Tet-Off lentiviral vector, pF 7x tOp MCS RS PGK Hygro TetR VP16. Antibodies directed against TAK1 (4505), caspase 8 (4927), caspase 3 (9661), PARP (9542), p-p65 (3033), IkBα (9242), p-cjun (9261), c-jun (9165), jnk (9252), p-jnk (9255), IKK2 (2684), p38 (9212), p-p38 (4511) all from Cell Signaling Technology, β-actin (Sigma: A-1978), p65 (SC-372), p-b-jun (SC-101724), b-jun (SC-46) from Santa Cruz, cFlip Dave-2 (ProScience: XA-1008), cIAP1 (Enzo Life Sciences: ALX-803-335-C100) and cIAP2 (homemade). We used in this study murine isoleucine zipper TRAIL where oligomerization of the ligand was promoted and stabilized by fusion of a isoleucine zipper motif to its amino terminus [27]. The irreversible and highly selective TAK1 inhibitor, 5Z-7-oxozeaenol was purchased from Bioaustralis Fine Chemicals. Human shRNA target for TAK1 was from Open Biosystems.

### Cell Culture, Lentiviral Infections, and Cell Treatments

HuH7, HT29, 293T cells were purchased from American Type Culture Collection and were maintained at 37°C, 10% CO_2_ in DMEM supplemented with 8% FBS, 2 mM L-glutamine, and penicillin/streptomycin.

To generate lentiviral particles, 293T cells were transfected using effectene (QIAGEN) with packaging constructs pCMV δR 8∶2, VSVg, and the relevant lentiviral plasmid in the ratio 1∶0.4∶0.6. After 24–48 h, the virus-containing supernatants were harvested and filtered. 12 µg/ml Polybrene was added and target cells were infected with virus supernatant 24–48 h. Selection was performed with 100–300 µg/ml hygromycin B and 2–5 µg/ml puromycin [28].

### Generation of TAK1−/− MEFs

MEFs were generated from E14 embryos from conditional knockout TAK1 mice[29] using standard procedures and infected with SV40 Large T antigen-expressing lentivirus (TAK1^flox/flox^ MEFs). To remove TAK1, the transformed TAK1^flox/flox^ MEFs were infected with a Cre-expressing lentivirus (pFU cre SV40 puro) and deletion was confirmed by PCR and Western Blot (TAK1^−/−^ MEFs).

### Cell Death Assays

Cells were seeded at 40% confluency and were allowed to adhere overnight. To evaluate survival if TRAIL-treated cells after inducing TAK1, TAK1 K63W, IKK2, FlipL, Flipp43, FlipR in TAK1^−/−^ MEFs with the inducible lentiviral system, we preincubated cells with 10 nM 4-hydroxytamoxifen (4HT) for 16 h and then added TRAIL (1 µg/ml) to cells for 24–48 h. Cell death was measured by propidium iodide staining and flow cytometry. MTT cell viability assays were performed in parallel with PI exclusion experiments.

### Western Blotting

Cells were washed in PBS and lysed in DISC buffer (1% Triton X-100, 10% glycerol, 150 mM NaCl, 20 mM Tris, pH 7.5, 2 mM EDTA and complete protease inhibitor cocktail (Roche) for 45 min at 4°C. Cell lysate was centrifugated at 14,000 g for 10 min and after addition of loading buffer to the supernatant (1% SDS and β-mercaptoethanol), boiled for 5 min. Samples were electrophoresed on 4–12% polyacrylamide gels (Invitrogen) and transferred to nitrocellulose membranes for antibody detection. The blots were stained with Ponceau S to confirm the uniformity of protein loading in each lane. All membrane-blocking steps and antibody dilutions were performed with 5% skin milk in PBST (PBS containing 0.1% Tween 20), and washing steps were performed with PBST. Proteins were visualized by ECL (GE Healthcare) after incubation of membranes with the corresponding HRP-coupled secondary antibody.

### Preparation of cDNA and Quantitative PCR

Total RNA isolation from MEFs was performed using Trizol Reagent (Invitrogen). Complementary DNA (cDNA) was synthesized from 4 µg of total RNA using Superscript III reverse transcriptase (Invitrogen) as per the manufacturer's protocol. Quantitative PCR was performed in a Light Cycler 480 instrument using the FastStart Universal SYBR Green Master (Roche). Each reaction was run in triplicate and the threshold (*C*_p_) values for each mRNA were subtracted from that of 18S ribosome mRNA, averaged, and converted from log-linear to linear term. PCR primers for each target gene: cIAP2 Forward 5′- GGAAATTGACCCTGCGTTATACAGA -3′, Reverse 5′-TCTCGGTCCATACACACTTTACACATT-3′, A20 Forward 5′-ACTTGGTCAAAATGGCATCA-3′, Reverse 5′-GCAGATAAATCCCACCCACT-3′, cFlipL Forward 5′-CCTCCAGCTCATCCTCTGTG-3′, Reverse 5′-TTTGTCCATGAGTTCAACGTG-3′ and as housekeeping gene: 18S Ribosome Forward 5′-TTGGAGGGCAAGTCTGGTG-3′, Reverse 5′-CCGCTCCCAAGATCCAACTA-3′.

PCR products amplified from RNA were analyzed by agarose gel electrophoresis to confirm that the PCR products were the correct length (data not shown).

## Results

### The Kinase Activity of TAK1 Is Required for MEFs to Survive Treatment with TRAIL

**Figure 1:**
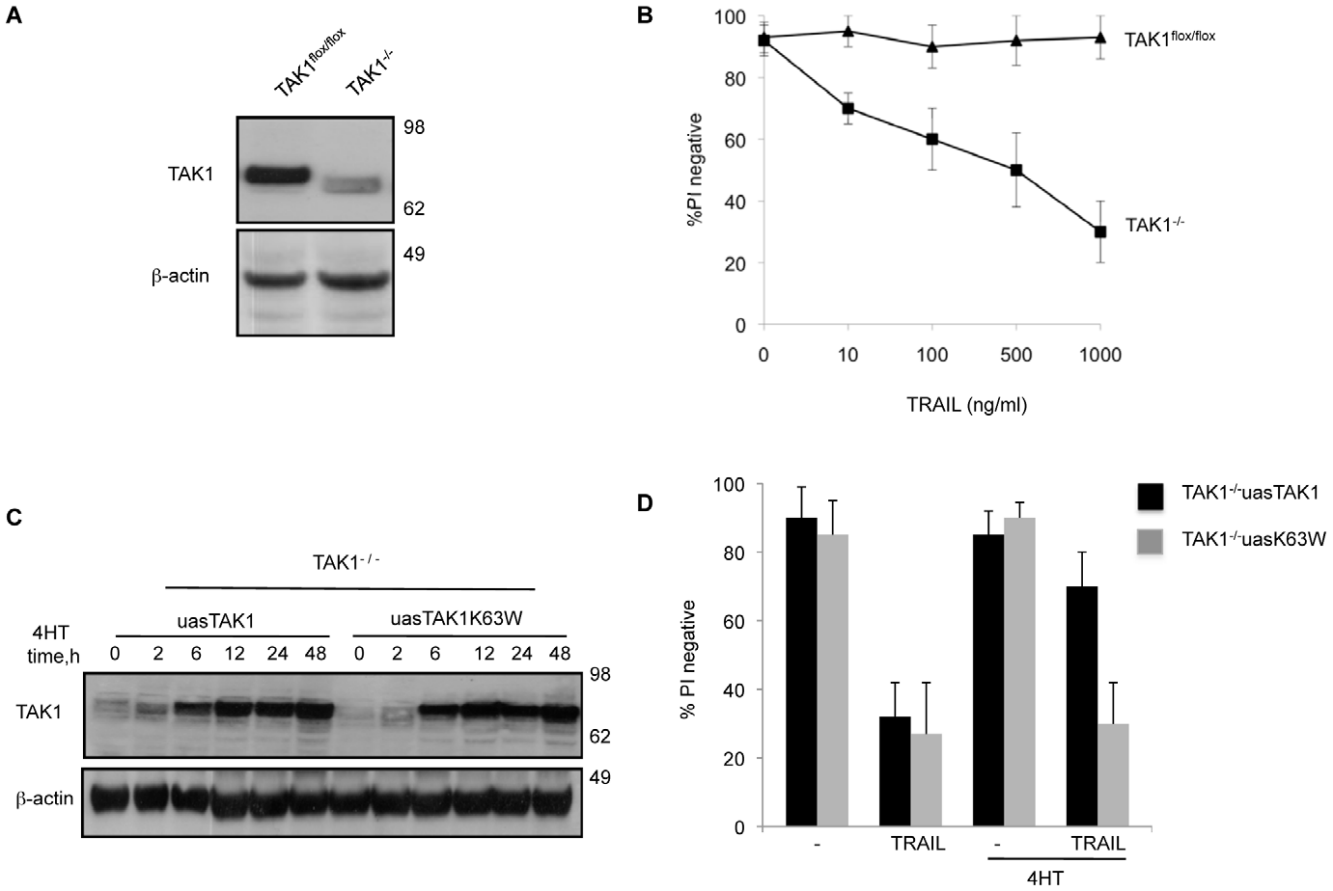
The kinase activity of TAK1 is required for TRAIL survival in MEFs. (A) Immunoblot analysis of TAK1 levels before and after TAK1^flox/flox^ MEFs were infected with lentivirus expressing Cre protein. (B) Polyclonal populations of wild-type (TAK1^flox/flox^) and TAK1 knock out (TAK1^−/−^) MEFs were treated with different concentrations of TRAIL for 48 h. (C,D). WT TAK1 but not mutant TAK1 (K63W, kinase null) complementation of TAK1^−/−^ MEFs protects against TRAIL sensitivity. (C) Time course of TAK1 levels after induction with 10 nM of 4-hydroxytamoxifen (4HT). (D) Cells were treated with TRAIL 1 µg/ml for 48 h and viability was assessed by PI staining and flow cytometry. The mean and SEM of three independent experiments is shown.

MEFs from mice in which exon 2 of the TAK1 gene is flanked by lox sequences (TAK1^flox/flox^) [29] were isolated and immortalized with SV40T. Subsequently, we used a lentiviral vector encoding Cre recombinase to generate TAK1−/− cells in vitro (Fig. 1A). Primary TAK1^flox/flox^ MEFs were also infected with Cre recombinase protein but TAK1 deleted primary MEFs failed to grow.

Whereas TAK1^flox/flox^ MEFs survived 48 h treatment with increasing concentrations of TRAIL, dose-dependent death was observed in TAK1^−/−^ MEFs (Fig. 1B). To confirm that the sensitivity of TAK1^−/−^ MEFs to TRAIL killing was due the loss of TAK1 alone, we reintroduced TAK1, or the kinase inactive mutant TAK1-K63W[30], into TAK1 knock out MEFs using a tamoxifen-inducible lentiviral system. As shown in Fig. 1C, the levels of TAK1 and TAK1-K63W increased following induction with tamoxifen. Complementation of TAK1 in TAK1^−/−^ MEFs effectively protected against TRAIL killing, whereas expression of TAK1-K63W failed to block TRAIL induced death as measured by PI exclusion (Fig. 1D) and MTT cell viability assay (Fig. S1).

In order to determine whether TAK1 deletion could induce TRAIL sensitivity in other cell types, two human cancer cell lines, HT29 and HuH7, were stably infected with lentivirus encoding shRNA against TAK1. The depletion of TAK1 (Fig. S1), sensitized HT29 cells to a greater extent than HuH7 cells to TRAIL (Fig. S1). Lower efficiency of shRNA mediated TAK1 knockdown in HuH7 cells may explain this lower sensitization to TRAIL.

In contrast to TRAIL, TAK1 deletion did not alter sensitivity to killing by FasL (Fig. S1).

### TRAIL-Induced Death of TAK1 Deleted Cells Depends on Caspase 8

**Figure 2:**
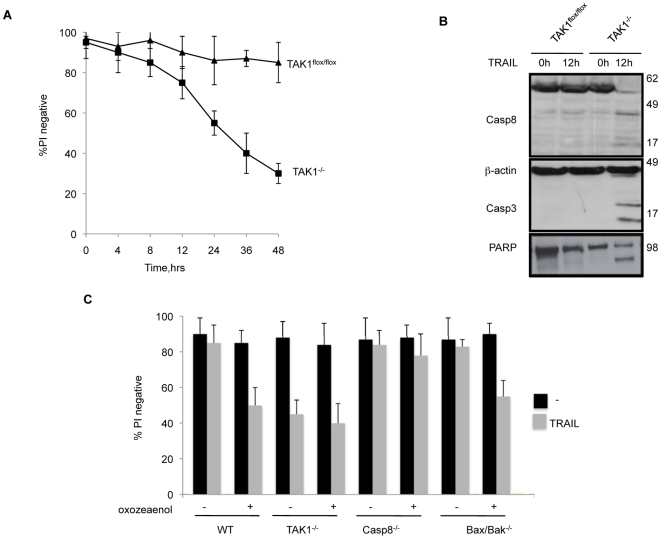
TRAIL induced death of TAK1^−/−^ MEFs is mediated by caspase 8. (A) Time course of viability of TAK1^flox/flox^ and TAK1^−/−^ MEFs exposed to TRAIL 1 µg/ml. (B) Cleavage of caspase 8, 3 and PARP in TRAIL treated WT and TAK1^−/−^ MEFs. (C) Wild-type, TAK1^−/−^, caspase 8 knock out (Casp8^−/−^), Bax and Bak double knock out (Bax^−/−^/Bak^−/−^) MEFs were coincubated with 30 nM 5Z-7-oxozeanol (an inhibitor of TAK1's catalytic activity) and TRAIL 1 µg/ml for 24 h. The mean and SEM of three independent experiments is shown.

TAK1^−/−^ MEFs began to die 12–14 h after TRAIL addition (Fig. 2A), and this was preceded by cleavage of caspase 8, 3 and PARP (Fig. 2B).

To assess whether a Bax/Bak or caspase 8 dependent apoptotic pathway was required for death of TAK1^−/−^ MEFs caused by TRAIL, we used 5Z-7-oxozeanol, a selective inhibitor of TAK1[31], [32], in caspase 8[33] and Bax/Bak knockout MEFs. Because TAK1^flox/flox^ cells treated with 5z-7-oxozeanol plus TRAIL succumbed at a similar rate to TAK1^−/−^ MEFs treated with TRAIL, and the addition of 5z-7-oxozeanol to TAK1^−/−^ MEFs did not cause an increase in cell death beyond that caused by TRAIL alone, at the concentrations used, 5z-7-oxozeanol does not appear to have any off-target effects (Fig. 2C).

Caspase 8^−/−^ MEFs were completely resistant to the treatment with 5z-7-oxozeanol plus TRAIL whereas Bax/Bak double knockout MEFs were efficiently killed by this combination. These results showed that the caspase 8 dependent apoptotic pathway, and not the Bax/Bak pathway, is responsible for death of TAK1^−/−^ MEFs caused by TRAIL.

### TAK1 Is Required for Activation of NF-κB and JNK Signalling Pathways by TRAIL

TAK1 is essential for NF-κB and JNK activation when cells are treated with TNF-α, TGF-β, TLR and IL-1[22], [23], [34]. As there have been several reports of NF-κB, JNK and p38 activation by TRAIL [1], we decided to study the role of TAK1 in the activation of these signalling pathways after TRAIL treatment.

**Figure 3:**
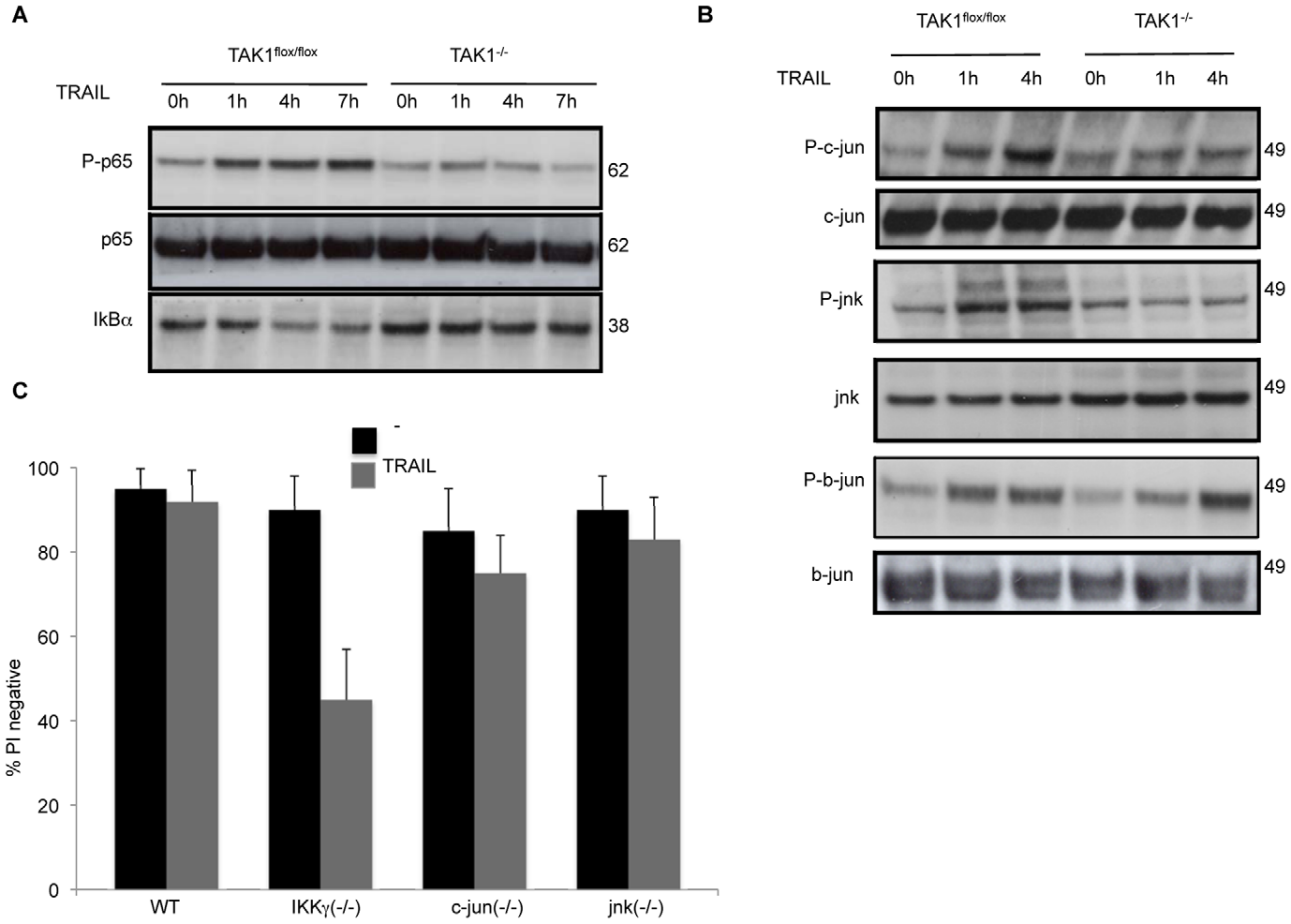
In TAK1^−/−^ MEFs, TRAIL fails to activate p65/RelA NF-κB and JNK signalling pathways. MEFs were stimulated with 1 µg/ml TRAIL for the indicated times, and cell lysates were probed for (A), phospho p65, p65, and IκB-α to determine NF-κB activation, and (B), phospho c-jun, JNK and b-jun to determine JNK activation. (C) Viability of Nemo/IKKγ knock out MEFs (IKKγ^−/−^), c-jun knock out MEFs (c-jun^−/−^) and JNK knock out MEFs (JNK^−/−^) after TRAIL exposure (1 µg/ml, 48 h). The mean and SEM of three independent experiments is shown.

TRAIL induced an increase in the phosphorylation of p65/RelA NF-κB that peaked at 7 h, and was accompanied by the degradation of IκBα in TAK1^flox/flox^ MEFs (Fig. 3A). However, phosphorylation of p65 and IκBα degradation were greatly impaired in TAK1 knockout MEFs (Fig. 3A).

TRAIL also caused JNK activation, as indicated by an increase in the phosphorylation of both JNK and c-jun in WT MEFs, whereas in the absence of TAK1 this was abolished (Fig. 3B). Moreover, b-jun, a member of the JUN family whose activation is not regulated by JNK, was phosphorylated in both TAK1^flox/flox^ and TAK1^−/−^ MEFs in response to TRAIL (Fig. 3B), indicating that TAK1 is not required for b-jun activation.

We also evaluated the kinetics of p38 MAPK activation in response to TRAIL by immunoblot. However, neither short nor long-term exposure of TAK1^flox/flox^ MEFs to TRAIL was able to activate p38 pathway (data not shown).

In order to distinguish the relative importance of NF-κB versus JNK activation in cell survival after TRAIL treatment, we tested the sensitivity of IKKγ, c-jun and jnk knockout MEFs to TRAIL (Fig. 3C). Interestingly, only IKKγ^−/−^ MEFs were sensitized to TRAIL-induced cytotoxicity, indicating that NF-κB, but not JNK pathway, plays a key role in protecting MEFs against killing by TRAIL.

### Activation of NF-κB in TAK1−/− MEFs Protects Against Killing by TRAIL

To test wether activation of NF-κB was sufficient to protect TAK1^−/−^ MEFs against TRAIL induced cell death, we infected TAK1^−/−^ MEFs with IKK2EE tamoxifen-inducible lentivirus (TAK1^−/−^uasIKK2EE). IKK2EE is an active mutant of IKK2 (S177E, S181E) that displays constitutive IKBα kinase activity and activates NF-κB[35].

**Figure 4:**
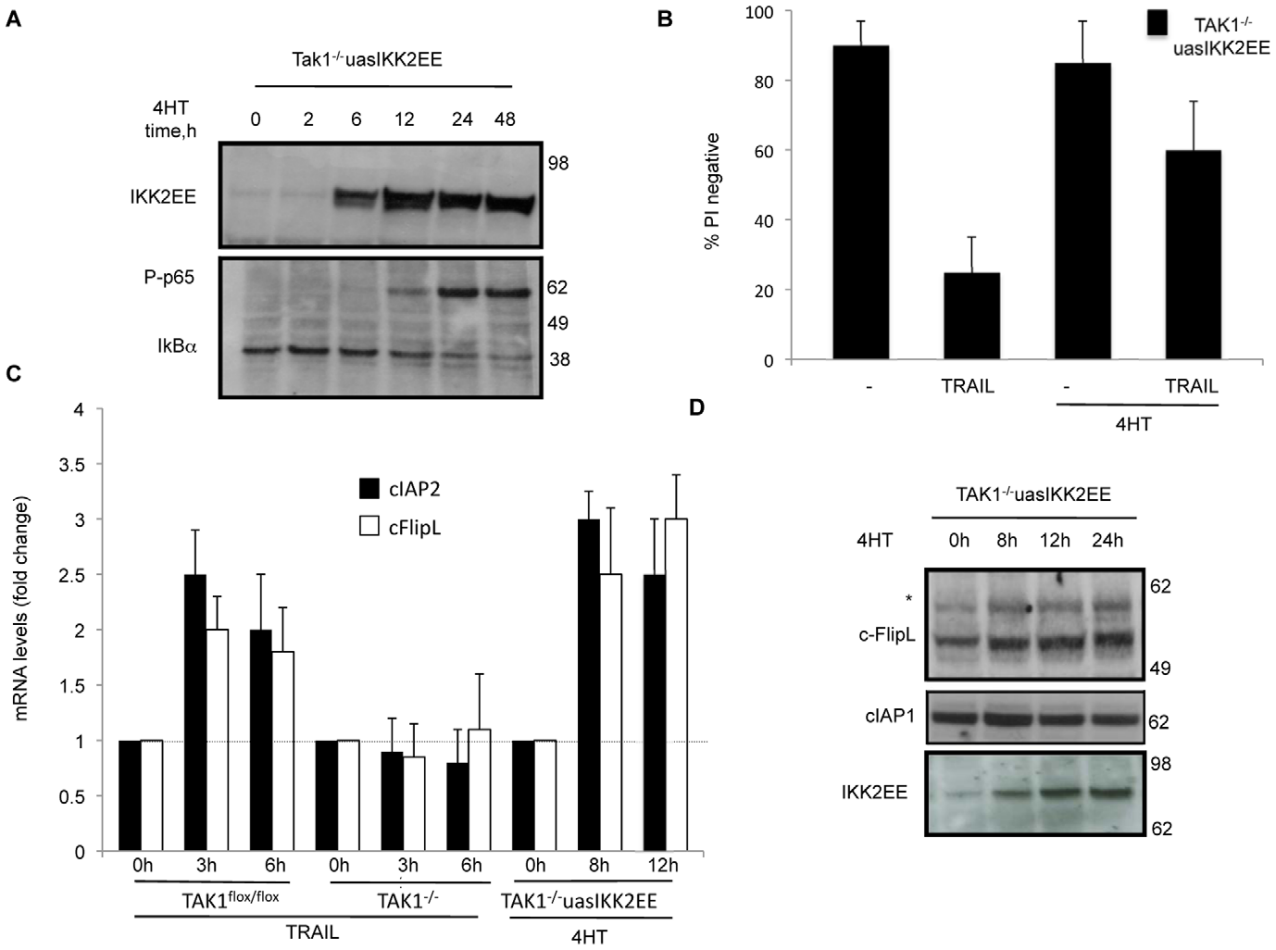
Activation of NF-κB by over-expression of IKK2-EE induces cFlip and rescues TAK1^−/−^ MEFs from TRAIL induced cell death. Inducible over-expression of dominant active IKK2-EE in TAK1^−/−^ MEFs was accompanied by an increase in phosphorylation of p65 and IκB-α degradation (A), and blocked sensitivity to TRAIL (1 µg/ml, 48 h) (B). Expression of both cIAP2 and cFlipL genes was elevated as determined by real time RT-PCR, using the housekeeping gene 18S rRNA as internal control (C). Western blot for cFlip and cIAP1 in TAK1^−/−^ MEFs stably infected with IKK2EE (TAK1^−/−^uasIKK2EE) after addition of 10 nM 4HT for different times (* indicates non-specific band).

Expression of IKK2EE in TAK1^−/−^ MEFs was detectable 6 h after addition of tamoxifen and was accompanied by an increase in both p65 phosphorylation and IkBα degradation (Fig. 4A). Moreover, the activation of NF-κB in TAK1^−/−^ MEFs protected cells against TRAIL induced cell death measured by PI exclusion (Fig. 4B) and an MTT cell viability assay (Fig. S2).

NF-κB can induce expression of several pro-survival genes, such as cIAP2, cFlip and A20, so we measured the levels of mRNA from these genes after TRAIL addition by quantitative real time RT-PCR.

TRAIL treatment of both TAK1^flox/flox^ and TAK1^−/−^ MEFs produced no change in A20 gene expression (data not shown). However as shown in Fig. 4C, TRAIL induced an increase in mRNA levels of cIAP2 and cFlipL, but this did not occur in TAK1 knockout MEFs. Importantly, activation of NF-κB in TAK1^−/−^ MEFs was sufficient to increase the mRNA levels of cIAP2 and FlipL (Fig. 4C), whereas A20 levels remained constant.

**Figure 5:**
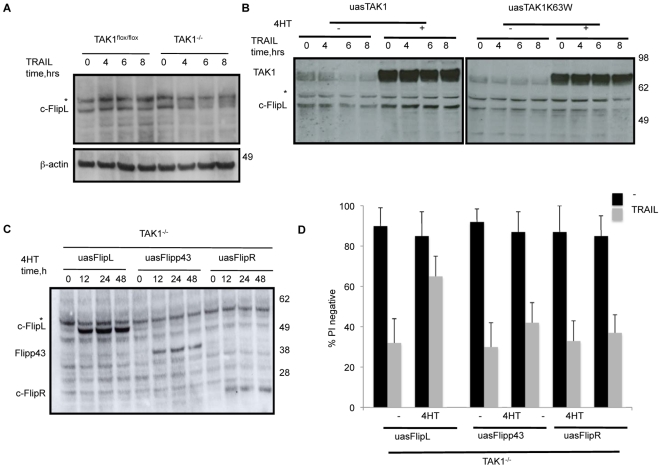
c-FlipL over-expression is able to reduce killing of TAK1^−/−^ MEFs by TRAIL, whereas Flipp43 and FlipR have little effect. (A) Endogenous c-Flip levels after TRAIL treatment in both TAK1^−/−^, TAK1^flox/flox^ MEFs and (B) TAK1^−/−^ MEFs reconstituted with TAK1 WT or TAK1 (k63W). (C) Lentiviral-mediated inducible expression of the different murine forms of c-Flip (c-FlipL, c-Flipp43, c-FlipR) in TAK1^−/−^ MEFs measured by Western blot. (D) The ability of the different forms of c-Flip to protect TAK1^−/−^ MEFs against killing by TRAIL (1 µg/ml, 48 h) was evaluated by PI exclusion.

Immunoblot analysis revealed that cFLipL protein level increased in TAK1^flox/flox^ MEFs upon treatment of TRAIL (Fig. 5A). In contrast, in MEFs lacking TAK1, cFlipL failed to increase after TRAIL addition, but appeared to decrease. Moreover, complementation of TAK1^−/−^ MEFs with WT, but not kinase mutant (K63W), TAK1 was able to restore the ability of TRAIL to induce an increase in cFlipL (Fig. 5B). Consistent with the changes in mRNA levels, the amount of cFlipL protein also increased after IKK2EE was induced in the TAK1^−/−^ MEFs (Fig. 4D). The presence of an upper non-specific band (marked with an asterisk) was consistently observed in all the cFlip blots.

Due to the importance of cIAP1 in TNF signalling [28], we also determined the levels of cIAP1 protein in both TAK1^flox/flox^ and TAK1^−/−^ MEFs in response to TRAIL (not shown) or after inducing NF-κB in TAK1 knockout MEFs (Fig. 4D), but no change was observed.

### cFLIPL but Not cIAP2 Overexpression in TAK1−/− MEFs Is Able to Inhibit TRAIL-Induced Cell Death

To discern the role of cIAP2 and cFlip in resistance to TRAIL killing, we over-expressed both proteins in TAK1^−/−^ MEFs and examined their effect on viability of cells treated with TRAIL.

First, we cloned all murine cFlip forms: cFlipL, cFlipp43 and cFlipR, into the tamoxifen-inducible lentiviral system, and infected Flip^−/−^ MEFs. Induction of cFlipL and cFlipp43 in cFlip^−/−^ MEFs caused an increase in the abundance of cFlipp43 and a novel cFlip cleaved variant respectively (Fig. S2, indicated by an asterisk), probably as an over-expression artefact, but caused no changes in cellular appearance or growth rate.

Compared to WT MEFs, Flip^−/−^ MEFs were extremely sensitive to killing by TRAIL, and induction of cFlipL and cFlipR significantly reduced cell death caused by TRAIL (Fig. S2).

However, when we introduced the different forms of cFlip into TAK1^−/−^ MEFs and induced their expression with tamoxifen (Fig. 5C), only cFlipL conferred resistance to death of TAK1^−/−^ cells in response to TRAIL (Fig. 5D).

**Figure 6:**
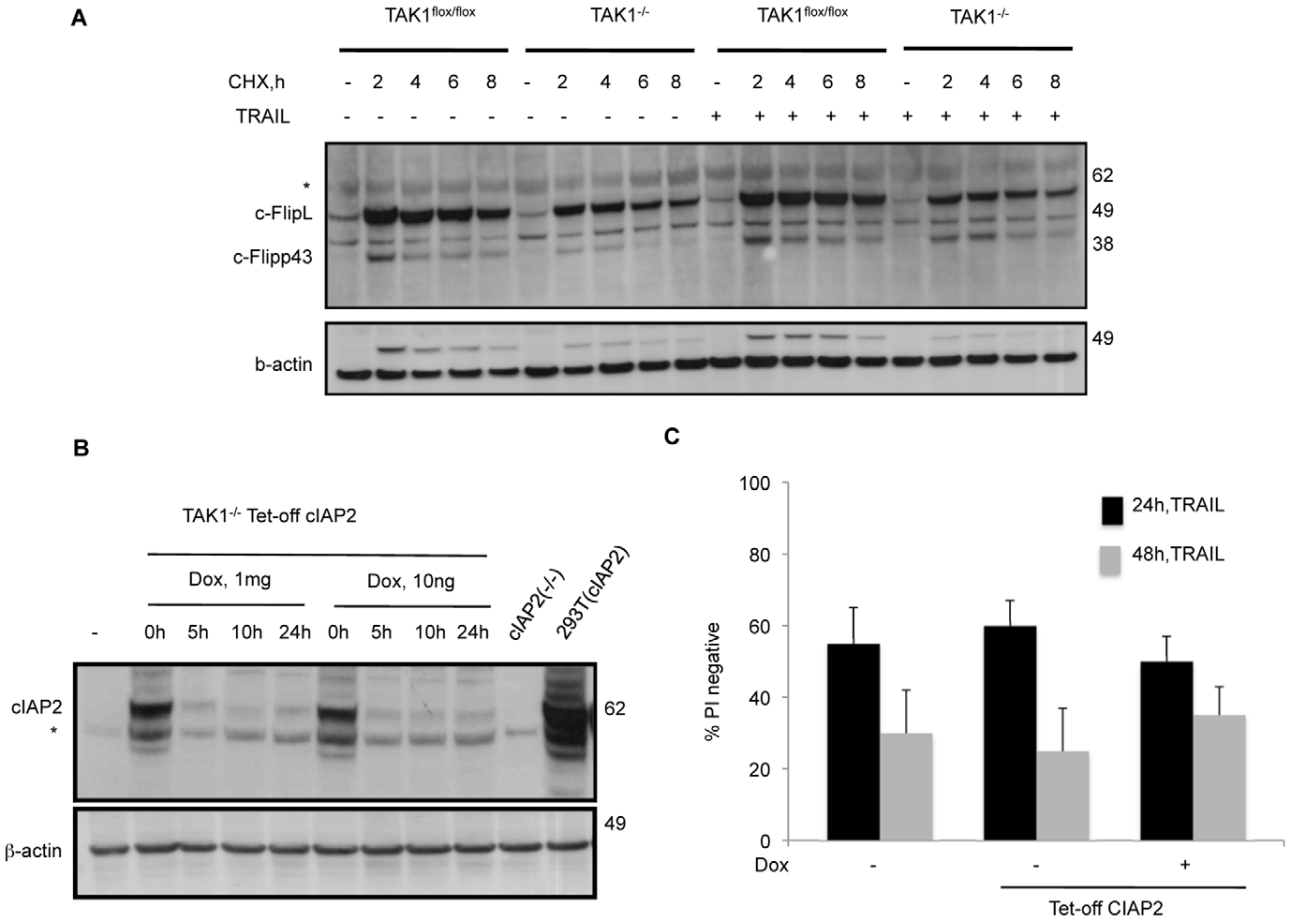
Cellular FlipL turnover is not affected by the presence or absence of TAK1, and cIAP2 over-expression in TAK1^−/−^ cells fails to protect against TRAIL killing. (A) TAK1^flox/flox^ and TAK1^−/−^ MEFs over-expressing cFlipL were incubated with 10 µg/ml cyclohexamide for different times and kinetics of cFlipL degradation was detected by immunoblot. (B,C) TAK1^−/−^ MEFs were stably infected with cIAP2 using a lentiviral Tet-off system. After addition of 1 µg/ml-10 ng/ml doxycycline cIAP2 protein levels were determined by Western blot (B), and cell viability in response to TRAIL was measured by PI exclusion (C).

To determine if this protection by cFlipL was due a change in protein half-life due to the absence of TAK1, we induced cFlipL in both wild type and TAK1 knock out MEFs, and incubated them with cyclohexamide with or without TRAIL. As shown in Fig. 6A, time-dependent degradation in cFlipL was similar in both MEFs, indicating that the cFlipL turnover was not significantly affected by the presence or absence of TAK1.

To see if the increase of cIAP2 gene expression observed in response to TRAIL (Fig. 4C) played any role in cell survival, TAK1 ^−/−^ MEFs were infected with a Tet-Off cIAP2 expressing lentivirus. Expression of high levels of cIAP2 in TAK1 ^−/−^ MEFs were achieved with this system and the presence of doxycycline (1 µg or 10 ng) effectively turned off cIAP2 induction (Fig. 6B). Interestingly, ectopic expression of cIAP2 in TAK1 ^−/−^ MEFs did not alter their sensitivity to TRAIL (Fig. 6C). Note however, we were unable to detect endogenous cIAP2 in MEFs by Western blot (Fig. 6B), suggesting MEFs usually only bear low basal levels of cIAP2.

## Discussion

NF-κB and JNK participate in a wide variety of cellular processes, including immunoregulation, inflammation, cell growth, cell differentiation and cell death. Because both induction of NF-κB and JNK activation by TRAIL were abolished in TAK1 deficient MEFs, and this corresponded to an increase in sensitivity to TRAIL induced apoptosis, we wanted to determine the role of these signalling pathways in allowing cell survival in the presence of TRAIL.

Our results showed that MEFs lacking genes for two key members of the JNK signalling pathway, c-jun and JNK, were not sensitive to killing by TRAIL, consistent with reports from other groups [36], [37].

TRAIL is mainly known for its ability to cause apoptosis, rather than for its ability to activate signal transduction by transcription factors such as NF-κB. Nevertheless, it has been shown that NF-κB is activated by the receptor for TRAIL, albeit more slowly and to a lesser extent that that caused by binding of TNF to TNFR1 [14].

Unlike WT MEFs, both TAK1^−/−^ MEFs and IKKγ^−/−^ MEFs were very sensitive to killing by TRAIL. Because activation of NF-κB by expression of constitutively active IKK2EE could protect the TAK1 knock out MEFs, activation of NF-κB is both necessary and sufficient to allow survival of TRAIL treated cells. Consistent with this, deletion of IKKγ in mouse hepatocytes caused hypersensitivity to TRAIL, resulting in massive liver damage [38].

Although genetic knockdown and chemical inhibition of TAK1 has been shown to enhance TRAIL-induced apoptosis in a cervical cancer cell line, the mechanism responsible for this sensitization was not described [25]. While preparing this manuscript, two independent groups have proposed different mechanisms to explain the role of TAK1 in TRAIL killing [24], [26]. Herrero-Martin et al 2009 concluded that TAK1 induces cytoprotective autophagy in TRAIL-treated breast epithelial cells. In MEFs, we did not observe any sign of autophagy following TRAIL treatment. In fact, caspase 8 knock out MEFs were totally resistant to TRAIL induced death, even when TAK1 was inhibited, indicating that in the absence of TAK1, TRAIL causes death in a caspase 8 dependent manner.

On the other hand, Morioka et al. 2009 [24] showed that deletion of TAK1 in mouse keratinocytes sensitized to TRAIL by ROS-dependent down-regulation of cIAP2, rather than acting via NF-κB. This might be due to a difference in cell type, because our results show that for MEFs to survive TRAIL treatment, NF-κB must activate and drive expression of cFlipL, which fails to occur in TAK1 knock out MEFs.

Interestingly, it has been reported that reduction of cFlipL and cIAP2 protein levels by c-myc is a major determinant of TRAIL sensitivity[39]. We over-expressed cIAP2 in TAK1 knock out cells using a lentiviral Tet-Off system. This approach allowed us to mimic the model of Morioka et al. model because in the presence of doxycycline cIAP2 expression was shut down. Unlike cFLip expression, cIAP2 over-expression in TAK1^−/−^ MEFs did not protect against TRAIL. Thus, the downstream signalling pathways activated by TAK1 in response to TRAIL may vary, depending on the cell type.

In some cells levels of cFlip may determine resistance to TRAIL induced apoptosis, because cFlip binds to the DISC complex and can prevent the recruitment and activation of procaspase 8. Although cFlip is constitutively expressed in normal cells, it is highly expressed in human cancer and thus is implicated in tumorigenesis [5], [8].

cFlip knock out MEFs were extremely sensitive to TRAIL induced cell death. The protection against TRAIL killing afforded by reconstitution of Flip^−/−^ MEFs with the two isoforms of cFlip, cFlipL and cFlipR, demonstrated their anti-apoptotic function against TRAIL. In agreement with these results, both c-FlipL and cFlipS are highly expressed in TRAIL-resistant as compared with TRAIL-sensitive human pancreatic cancer cells [13]. Furthermore, erythroid differentiation sensitizes leukaemia cells to TRAIL killing by downregulation of both c-Flip splicing isoforms [40].

However, only expression of FlipL, and not the other Flip forms, was able to efficiently block TRAIL killing in TAK1 knock out MEFs. Accordingly, the protective role of cFlipL in response to death ligands has been observed in both over-expression and loss-of-function studies[11], [41].

c-Flip expression is carefully regulated at different levels. In addition to regulation of gene transcription, turnover of c-Flip protein is actively controlled by ubiquitin signalled degradation [42]. In fact, JNK activation during TNF signalling has been proposed to promote the proteosomal degradation of c-FlipL. Although loss of TAK1 resulted in inability of TRAIL to activate JNK, the half-life of cFlipL was similar in the presence or absence of TAK1. These observations suggest that modulation of cFlipL's stability is not involved in its capacity to inhibit TRAIL killing in TAK1^−/−^ MEFs.

Here we report that TAK1 is essential for TRAIL to induce NF-κB and JNK activation. Moreover, NF-κB activation can overcome the sensitivity of TAK1 knock out MEFs to killing by TRAIL. Finally, cFlipL but not another NF-κB dependent antiapoptotic regulator, cIAP2, can protect TAK1^−/−^ MEFs from killing by TRAIL.

In conclusion, inhibition or deletion of TAK1 led to impaired NF-κB dependent cFlipL expression, allowing caspase 8 activation and cell death in response to TRAIL. Thus, NF-κB may negatively regulate TRAIL induced cell death in TAK1 knock out MEFs by increasing cFlipL levels. Compounds that inhibit TAK1 may increase the range of tumor cells that are sensitive to TRAIL.

## Supporting Information

Figure S1 TAK1 down-regulation sensitizes HT29 and HuH7 cancer cell lines to tumor necrosis factor-related apoptosis-inducing ligand (TRAIL). HT29 and HuH7 cells were infected with a lentivirus expressing TAK1 shRNA or scramble (control). (A,B) TAK1 levels were examined by western blot and sensitivity to TRAIL by PI exclusion. No differences in cell viability between TAK1^−/−^ and TAK1^flox/flox^ MEFs after Fas treatment. (C) Cells were incubated with Fas during 24 hours. (D) MTT results of the same experiment as Fig. 1D.
(0.53 MB TIF)

Figure S2 c-FlipL and c-FlipR isoforms are the main inhibitors of TRAIL-induced cell death. Flip knockout MEFs (Flip^−/−^) were complemented with FlipL (uasFlipL), Flipp43 (uasFlipp43), and FlipR (uasFlipR). (A) Cell survival was measured after treating them with TRAIL (1 µg/ml, 24 h). (B) Protein levels of the different forms of Flip were detected by immunoblot. (C) MTT cell viability assay corresponding to the same experiment as Fig. 4B.
(0.51 MB TIF)
