# Correction: TAK1 Is Required for Survival of Mouse Fibroblasts Treated with TRAIL, and Does So by NF-κB Dependent Induction of cFLIPL

**DOI:** 10.1371/annotation/4e35cd59-c68f-4020-addb-18eb896112c5

**Published:** 2010-12-03

**Authors:** Josep Maria Lluis, Ulrich Nachbur, Wendy Diane Cook, Ian Edward Gentle, Donia Moujalled, Maryline Moulin, Wendy Wei-Lynn Wong, Nufail Khan, Diep Chau, Bernard Andrew Callus, James Edward Vince, John Silke, David Lawrence Vaux

Although all the lanes in the actin blot from Figure 1C did come from the same gel, due to lack of annotations on the original files, the authors cannot be 100% certain they came from the same gel as the upper (TAK1) panel. Therefore, as an indication of the evenness of the loading of the lanes, the authors have provided a corrected Figure 1C, that shows more of the TAK1 blot, so that loading can be assessed from the cross-reactive bands at: http://www.plosone.org/co...

The authors incorrectly cropped the middle panel of Figure 2B included in the original article. See the corrected figure here: http://www.plosone.org/co...

Figure 5A: The authors would also like to provide some clarification regarding the data reported in Figure 5A. The c-FLIP-L bands and the beta-actin loading controls for this blot come from different gels. The loading controls were from the same samples run on a replicate gel, because as a result of repeated stripping and reprobing, the beta-actin probing of the original gel was unclear.

