# Supplementary figures and images for: TAK1 Is Required for Survival of Mouse Fibroblasts Treated with TRAIL, and Does So by NF-κB Dependent Induction of cFLIPL
Source: PLoS One. 2010 Jan 8;5(1):e8620. doi: 10.1371/journal.pone.0008620 (PMC2797639; doi:10.1371/journal.pone.0008620)

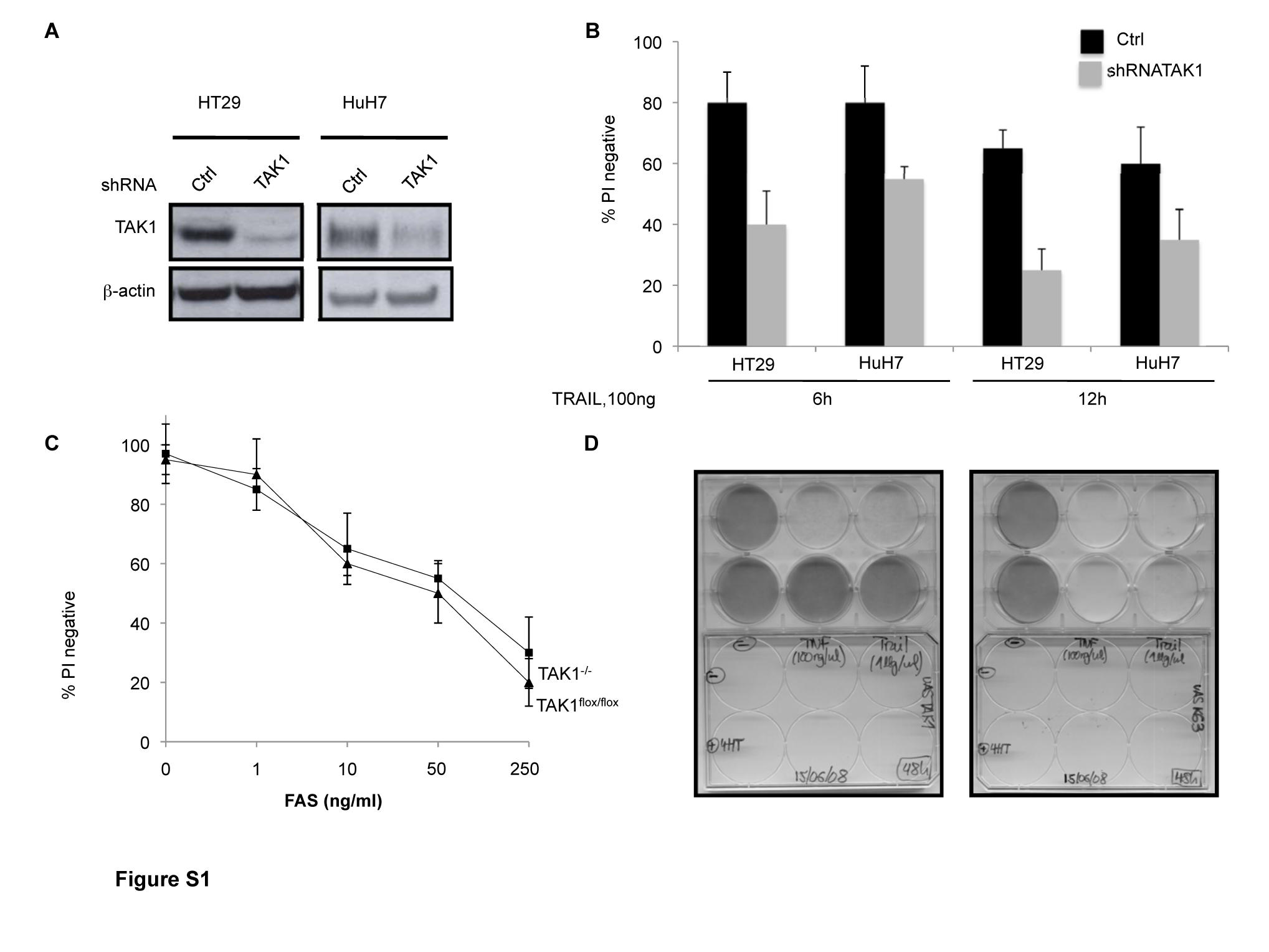

Supplement: Figure S1 — TAK1 down-regulation sensitizes HT29 and HuH7 cancer cell lines to tumor necrosis factor-related apoptosis-inducing ligand (TRAIL). HT29 and HuH7 cells were infected with a lentivirus expressing TAK1 shRNA or scramble (control). (A,B) TAK1 levels were examined by western blot and sensitivity to TRAIL by PI exclusion. No differences in cell viability between TAK1−/− and TAK1flox/flox MEFs after Fas treatment. (C) Cells were incubated with Fas during 24 hours. (D) MTT results of the same experiment as Fig. 1D. (0.53 MB TIF) [file pone.0008620.s001.tif]

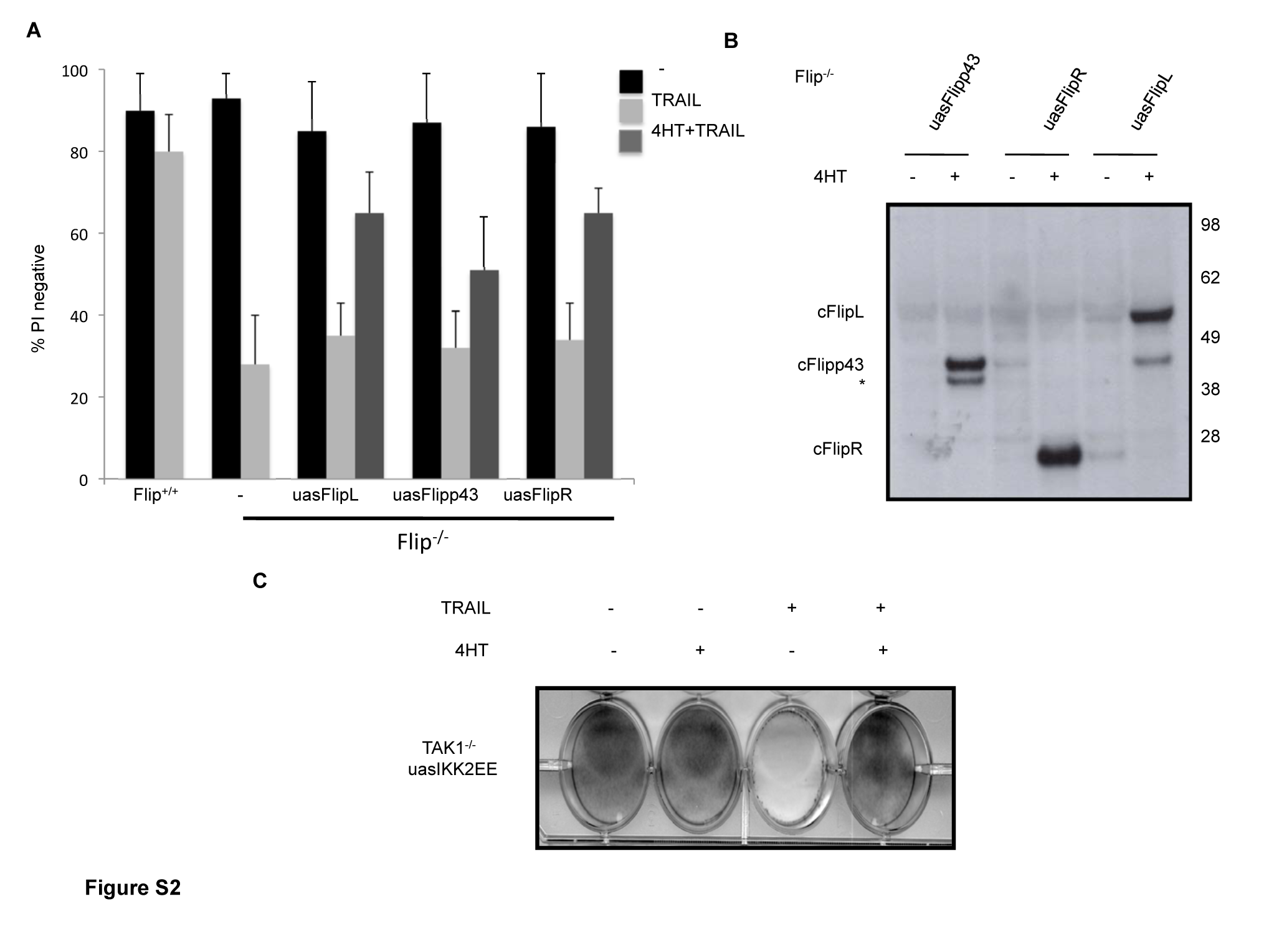

Supplement: Figure S2 — c-FlipL and c-FlipR isoforms are the main inhibitors of TRAIL-induced cell death. Flip knockout MEFs (Flip−/−) were complemented with FlipL (uasFlipL), Flipp43 (uasFlipp43), and FlipR (uasFlipR). (A) Cell survival was measured after treating them with TRAIL (1 µg/ml, 24 h). (B) Protein levels of the different forms of Flip were detected by immunoblot. (C) MTT cell viability assay corresponding to the same experiment as Fig. 4B. (0.51 MB TIF) [file pone.0008620.s002.tif]
